# Galangin Inhibits Gastric Cancer Growth Through Enhancing STAT3 Mediated ROS Production

**DOI:** 10.3389/fphar.2021.646628

**Published:** 2021-04-26

**Authors:** Xiaohui Liang, Ping Wang, Chun Yang, Fei Huang, Hui Wu, Hailian Shi, Xiaojun Wu

**Affiliations:** Shanghai Key Laboratory of Compound Chinese Medicines, The Ministry of Education (MOE) Key Laboratory for Standardization of Chinese Medicines, The SATCM Key Laboratory for New Resources and Quality Evaluation of Chinese Medicines, Institute of Chinese Materia Medica, Shanghai University of Traditional Chinese Medicine, Shanghai, China

**Keywords:** galangin, gastric cancer, apoptosis, proliferation, ROS, stat3

## Abstract

Galangin, a flavonoid isolated from the rhizome of *Alpinia officinarum* (Hance), exerts anticancer activities against many cancer cells such as liver cancer, breast cancer, lung cancer and esophageal cancer. However, the effect, as well as the underlying molecular mechanism of galangin on gastric cancer remains to be elucidated. In the present study, galangin inhibited cell viability of MGC 803 cells but not normal gastric mucosal epithelial GES-1 cells. It suppressed cell proliferation accompanied by reduced Ki67 and PCNA expression, promoted apoptosis shown by decreased Bcl-2 and elevated cleaved caspase-3 and cleaved PARP. And, galangin significantly inactivated JAK2/STAT3 pathway. When STAT3 was overexpressed, the proliferation inhibition and apoptosis promotion induced by galangin were abrogated. Meanwhile, galangin increased ROS accumulation, and reduced Nrf2 and NQO-1, but elevated HO-1 in MGC 803 cells. NAC, a ROS scavenger, rescued ROS over-accumulation and proliferation inhibition of galangin. STAT3 overexpression also counteracted excessive ROS accumulation induced by galangin. Consistent with the *in vitro* experiments, in nude mice exnografted with MGC 803 cells, galangin inhibited tumor growth and reversed the abnormally expressed proteins, such as p-JAK2, p-STAT3, Bcl-2, cleaved caspase-3, cleaved PARP, and Ki67. Taken together, galangin was suggested to inhibit the growth of MGC 803 cells through inducing apoptosis and decreasing cell proliferation, which might be mediated by modulating STAT3/ROS axis. Our findings implicate a potential application of galangin for gastric cancer therapy possibly with low toxicity.

## Introduction

Gastric cancer caused death ranks third among all cancer-related deaths worldwide, and the 5-year survival rate of cancer patients is still less than 5% (Digklia and Wagner, 2016; Wang et al., 2019). Surgery is currently considered to be the only radical treatment. However, chemotherapy almost runs through the treatment of middle- and late-stage gastric cancer after surgery (Amedei et al., 2011; Corso et al., 2013). Unfortunately, its side effects gravely reduce the life quality of cancer patients and limit its clinical efficacy as well. Furthermore, patients with gastric cancer in advanced stages often poorly respond to chemotherapy. Therefore, it is still urgent to develop new chemotherapeutic drug with low toxicity against gastric cancer.

Many oncogenic proteins involve in the cancer progression including signal transducer and activator of transcription 3 (STAT3), an oncogenic transcription factor, which participates in the cell apoptosis (Kim and Park, 2018; Wu et al., 2019), proliferation and autophagy (You et al., 2015; Furtek et al., 2016; Kim et al., 2017; Fathi et al., 2018; Li et al., 2018). Janus associated kinase (JAK)2, an important tyrosine kinase, belongs to the Janus family and plays a positive feedback regulatory role in the expression of STAT3 (Kaptein et al., 1996; Justicia et al., 2000). Suppression of JAK/STAT3 pathway, for instance, by piperlongumine, results in gastric cancer inhibition (Justicia et al., 2000; Song et al., 2016). Therefore, STAT3 should be a valuable target for cancer therapy.

Reactive oxygen species (ROS) is a double-edged sword for cancer cells, and plays different roles in different stages of cancer (Prasad et al., 2017). ROS promotes proliferation and growth of cancer cells through activating various cell signaling pathways, which are primarily mediated through the transcription factors nuclear factor-kappa B (NF-κB) and STAT3, hypoxia-inducible factor 1α (HIF1α), kinases, growth factors, cytokines and other proteins (Prasad et al., 2017). However, excessive accumulation of ROS can cause cell damage and apoptosis (Kudryavtseva et al., 2016; Ismail et al., 2019).

**FIGURE 1:**
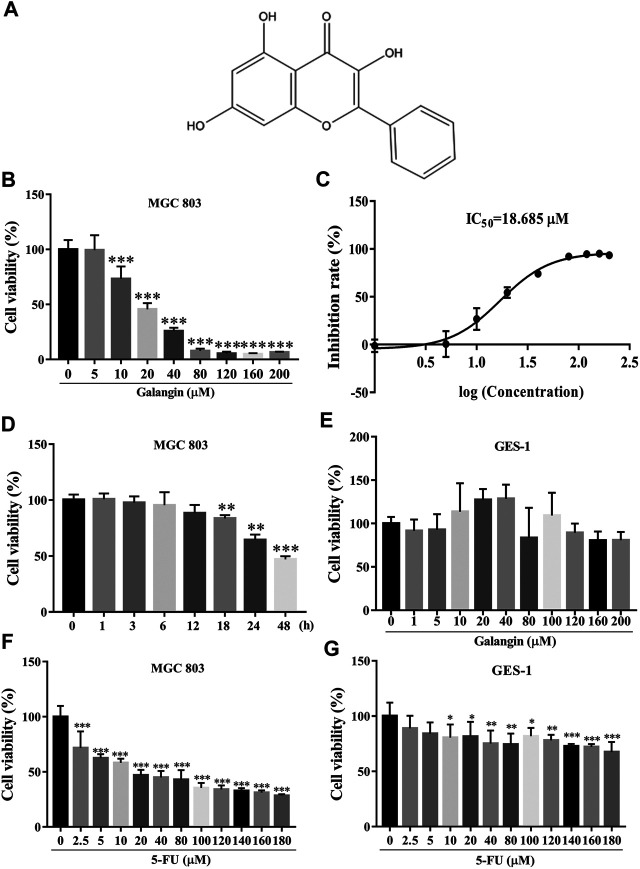
Galangin inhibits cell viability of MGC 803 cells. **(A)** Chemical structure of Galangin. **(B)** Galangin reduced cell viability of MGC 803 cells in a dose-dependent manner after treatment for 48 h. **(C)** IC_50_ of galangin for 48 h in MGC 803 cells. **(D)** Galangin (20 μM) inhibited cell viability of MGC 803 cells in a time-dependent manner. **(E)** Galangin showed no significant effect on GES-1 cells (48 h), a normal gastric mucosal epithelial cell line. **(F)** 5-FU inhibited cell viability of MGC 803 cells (48 h). **(G)** 5-FU inhibited cell viability of GES-1 cells (48 h). All of the data were shown as mean ± SD, and differences among ≥3 groups (Figures 1B,D,E) were analyzed via one-way ANOVA with Dunnett test by using GraphPad 7.0 software; *p < 0.05; **p < 0.01; ***p < 0.001, compared with control group. n ≥ 3.

Galangin, named as 3, 5, 7-trihydroxyflavone (Figure 1A), is a natural flavonoid compound, mainly present in the rhizome of *Alpinia officinarum* Hance (Zingiberaceae) (Liu et al., 2018). It showed anti-tumor activity against several cancer cells except gastric cancer, such as liver cancer (Zhang et al., 2010), breast cancer (Liu et al., 2018), lung cancer (Yu et al., 2018) and esophageal cancer (Ren et al., 2016) *in vitro*. In this study, the effect and the underlying molecular mechanism of galangin on gastric cancer cells were investigated, which may extend its potential clinical application.

## Materials and Methods

### Cell Culture

Gastric cancer cell line MGC 803 and mucosal epithelial cell line GES-1 were obtained from Cell Bank, Type Culture Collection of Chinese Academy of Sciences (Shanghai, China). The cells were cultured in RPMI 1640 medium (Meilunbio, Cat. No.: MA 0215) supplemented with 10% fetal bovine serum (FBS, Gibco, Cat. No.: 10099–141), and 1% penicillin and streptomycin (Meilunbio, Cat. No.:MA0110) in a humidified incubator with 5% CO_2_ at 37°C.

### Cell Viability Assay

After treatment with galangin at different concentrations for 0–48  h, the cells were incubated with 20 μL CCK-8 solution (Cell Counting Kit-8, DOJINDO Laboratories, Cat. No.: CK04) for another 1 h at 37°C. Absorbance of the medium was detected at 450 nm on a Thermo Scientific Varioskan Flash microplate reader (Thermo, United States). The cell viability rate was calculated as follows (absorbance of drug-treated sample/absorbance of control sample)  ×  100.

### Immunocytochemistry

MGC 803 cells were cultured on coverslips in 24-well plate for 12 h, followed by galangin treatment (20 μM) for 48 h. After washed once with 1 × PBS solution, the cells were fixed with 4% paraformaldehyde (PFA), permeabilized with 0.3% Triton-X-100 and blocked with 10% donkey serum. Then they were incubated with primary antibody against Ki67 (Cat. No. ab16667) overnight at 4°C. Then they were incubated with secondary antibody conjugated with Alexa-488 fluorophore for 1 h at room temperature (RT). After washed with 1 × PBS solution twice, the coverslips with cells were mounted on glass slides with mounting medium containing DAPI. Immunofluorescence images were acquired by an inverted fluorescence microscope (IX81, Olympus, Japan).

### Annexin V/PI Staining

After galangin treatment at 20 μM for 48 h, MGC 803 cells were harvested by trypsin without EDTA, and washed twice with 1 × PBS solution. Consequently, the cells were double stained with Annexin V/PI according to manufacturer’s protocol, and detected on a Guava flow cytometer (Guava easycyte HT, Millipore, Germany).

### Hoechst 33258 Staining

After galangin (20 μM) treatment for 48 h, MGC 803 cells were fixed with 4% PFA for 10 min. Then the cells were gently rinsed with 1 × PBS solution and stained with 10 μg/ml Hoechst 33258 solution for another 15 min. Finally, the cells were washed with 1 × PBS solution, and observed under a fluorescence microscope (IX81).

### EdU Staining

MGC 803 cells were plated at a density of 8.0 × 10^4^ cells/ml on a 96-well plate and allowed to adhere to plates overnight. EdU staining was carried out using the EdU imaging kit (RiboBio Co., China). Briefly, cells treated with galangin (20 μM) were first labeled with 50 μM EdU at 37°C for 2 h. Subsequently, they were fixed with 4% PFA for another 30 min, and incubated with 1 × PBS solution containing 0.5% Triton X-100 for 10 min. After washed with 1 × PBS solution, the cells were incubated with 100 μL dying solution for 30 min in the dark. Finally, the nuclei were stained with Hoechst 33342 solution for another 30 min. Fluorescent images were captured by fluorescence microscopy (IX81). Data were analyzed by using ImageJ software.

### Mitochondrial Membrane Potential Measurement

MMP was measured by using fluorescent probe JC-1 (Santa Cruz, Cat. No.: sc-364, 116). After galangin (20 μM) treatment for 24 and 48 h, MGC 803 cells were rinsed with 1×HBSS solution (Gibco, Cat. No.: 14025–092) and incubated with JC-1 (10 μM) at 37°C for another 30 min. After that, the cells were rinsed with 1 × HBSS solution once again, and the fluorescent intensity of the JC-1 monomers and aggregates was detected under different conditions (Ex (λ) 485 nm, Em (λ) 530 nm for monomers; Ex (λ) 530 nm, Em (λ) 590 nm for aggragates) on a microplate reader (Varioskan Flash, Thermo Scientific, United States). Fluorescent images were captured under a fluorescent microscope (IX81).

### Western Blotting Analysis

MGC 803 cells or tumor tissues were lyzed in CelLytic™ MT Cell Lysis Reagent (Sigma, Cat. No.: C3228) containing protease and phosphatase inhibitors (Roche, Cat. No.: 04693116001, 04906837001) for 30 min on ice. After centrifugation at 12,000 rpm at 4°C for 15  min, the supernatant was collected and the protein concentration was quantified by using BCA assay. Totally, 30 μg proteins from each samples were separated by SDS-PAGE (10%) and transferred onto PVDF membrane by wet transfer. Afterward, the membranes were blocked with 0.5% BSA solution for 1 h and incubated with primary antibodies against JAK2 (1:1,000, D2E12, #3230, CST, United States), p-JAK2 (1:1,000, C80C3, #3776, CST, United States), STAT3 (1:1,000, 124H6, #9139, CST, United States), p-STAT3 (1:2000, D3A7, #9145, CST, United States), Ki67 (1:500, sp6, #ab16667, Abcam, United States), Bcl-2 (1:1,000, 50E3, #2870, CST, United States), Bax (1:1,000, D2E11, #5023, CST, United States), Cleaved caspase-3 (1:1,000, 5A1E, #9664, CST, United States), Caspase-3 (1:1,000, D3R6Y #14220, CST, United States), LaminB1(1:3,000, # 6,581–1 Epitomics United States), Nrf2 (1:500, sc-722, Santa Cruz, United States), NQO1 (1:500, sc-32793, Santa Cruz, United States), HO-1 (1:500, sc136960, Santa Cruz, United States), PARP (1:1,000, 9532s, Santa Cruz, United States), and GAPDH (1:200,000, D16H11, #5174, CST, United States) overnight at 4°C. After washed with 1 ×  PBST, the membranes were incubated with respective secondary antibodies conjugated with horseradish peroxidase for another 1 h at RT. The protein bands were visualized with Immobilon™ Western Chemiluminescent HRP Substrate (Millipore Corporation, Cat. No.: WBKLS0500), and the images were taken under the visualization instrument Tanon-5200 (Tanon, China).

### Real-Time Quantitative PCR

Total RNA was isolated from the harvested MGC 803 cells by using TRIzol Reagent (Ambion, REF: 15596018). cDNA was reversely transcribed from RNA (2 μg) by using Revert Aid First Strand cDNA Synthesis Kit (Thermo, Cat. No.: K1622) according to the manufacturer’s protocol. Real-time quantitative PCR was performed with SYBR reagent (VazymE, L/N 7E141I7, Cat. No.: Q111–02) on Quant Studio six Flex System (Life technologies, Cat. No.: 20170777). Quantification of target genes was determined by the 2^−ΔΔCt^ method. And the relative expression of individual genes was normalized to that of GAPDH in the same sample. The sequences of the primers (GeneRay, China) used were listed in Table 1.

**TABLE 1:** Primers used in qPCR analysis.

Genes	Forward primer	Reverse primer
Ki67	5′-CAG​ACA​TCA​GGA​GAG​ACT​ACA​C-3′	5′-AAG​AAG​TTC​AGG​TAC​CTC​AGT​G-3′
PCNA	5′-TAA​TTT​CCT​GTG​CAA​AAG​ACG​G-3′	5′-AAG​AAG​TTC​AGG​TAC​CTC​AGT​G-3′
GAPDH	5′- GCA​CCG​TCA​AGG​CTG​AGA​AC-3′	5′- TGG​TGA​AGA​CGC​CAG​TGG​A-3′

### ROS Level Measurement

The intracellular ROS production in MGC 803 cells was measured by the oxidation-sensitive fluorescent probe 2′,7′-dichlorofluorescin diacetate (DCFH-DA). In brief, after galangin (20 μM) treatment for 12, 18, 24, and 48 h, MGC 803 cells were gently washed with 1 × HBSS solution and incubated with DCFH-DA (10 μM) for 30 min at 37°C. Fluorescence was immediately measured on a Varioskan Flash microplate reader (Ex (λ) 485 nm, Em (λ) 535 nm, Thermo, United States).

### STAT3 Transient Transfection

STAT3 was over-expressed by transiently transfecting p-CMV-STAT3 plasmid in MGC 803 cells. The cells are seeded in a medium dish until growing to 50% confluency, then transiently transfected with p-CMV-STAT3 plasmid or p-CMV plasmid for 24 h by using NEOFECT DNA transfection reagent (Neofect Beijing Biotech, China). After treatment with galangin (20 μM) for 48 h, MGC 803 cells were subjected to CCK-8 assay and western blotting assay.

### Animals and Treatments

Healthy 4-week-old male nude mice (12 ± 2 g) were obtained from Shanghai Slake Experimental Animal Co., Ltd. and kept under SPF animal rooms. All animal experiments were carried out in accordance with the protocol approved by the Animal Ethics Committee in Shanghai University of Traditional Chinese Medicine (SHUTCM), which complies with international rules and policies for laboratory animal use and care as found in the European Community guidelines (EEC Directive of 1986; 86/609/EEC). All animal experiments were approved by the institutional Ethics Committee of SHUTCM (PZSHUTCM200724009).

After one week habituation, the mice were inoculated subcutaneously with MGC 803 cells (5 × 10^6^ cells in 200 μL PBS per mouse). Body weight and tumor volume were measured every three days. When the tumor volume reached approximately 50 mm^3^, the mice were randomly divided into three groups, namely Control group, Galangin group and 5-Fluorouracil (5-FU) group. Galangin was dissolved in 0.5% sodium carboxylmethylcellulose. 5-Fu was dissolved in 1 × PBS solution. Control mice were intraperitoneally injected with 0.5% sodium carboxylmethylcellulose solution. Galangin-treated mice were administered with galangin (120 mg/kg) by oral gavage once a day. Meanwhile, 5-FU-treated mice were intraperitoneally injected with 5-FU (50 mg/kg) twice a week. Tumor volume was calculated according to the formula [length × (width)^2^]/2. Three weeks after treatment, all the nude mice were sacrificed, and the isolated tumors were weighted and then cut into several parts which were either fixed in 4% PFA or stored at −80°C for further analysis.

### Statistical Analysis

All of the data were presented as the mean ± standard deviation (SD). Differences between two groups were analyzed by the Student’s *t*-test. Differences among more than two groups were analyzed by one-way ANOVA with Dunnett or Tukey test using GraphPad 7.0 software (La Jolla, CA, United States). The value of *p* < 0.05 was considered to indicate a statistically significant difference.

## Results

### Galangin Reduced Cell Viability of MGC 803 Cells *In Vitro*

As shown in Figures 1B,C, galangin significantly reduced the cell viability of MGC 803 cells after treatment for 48 h. The IC_50_ value of galangin on MGC 803 cells for 48 h was 18.685 μM. Galangin inhibited cell viability of MGC 803 cells as early as 18 h after treatment. The cell viability of MGC 803 cells inhibited by galangin (20 μM) were 16.34, 35.57, and 52.97%, respectively, after treatment for 18, 24, and 48 h (Figure 1D). In contrast, after treatment for 48 h, galangin used below 200 μM had no significant cytotoxicity to GES-1 cells (Figure 1E), suggesting its low cytotoxicity to normal cells. 5-FU (≥2.5 μM) treatment for 48 h significantly inhibited cell viability of MGC 803 cells (Figure 1F), however, it (≥10 μM) also significantly suppressed cell viability of GES-1 cells after treatment for 48 h (Figure 1G). These results indicated that galangin inhibited gastric cancer cell viability with lower toxicity than 5-FU.

### Galangin Induced Apoptosis of MGC 803 Cells

**FIGURE 2:**
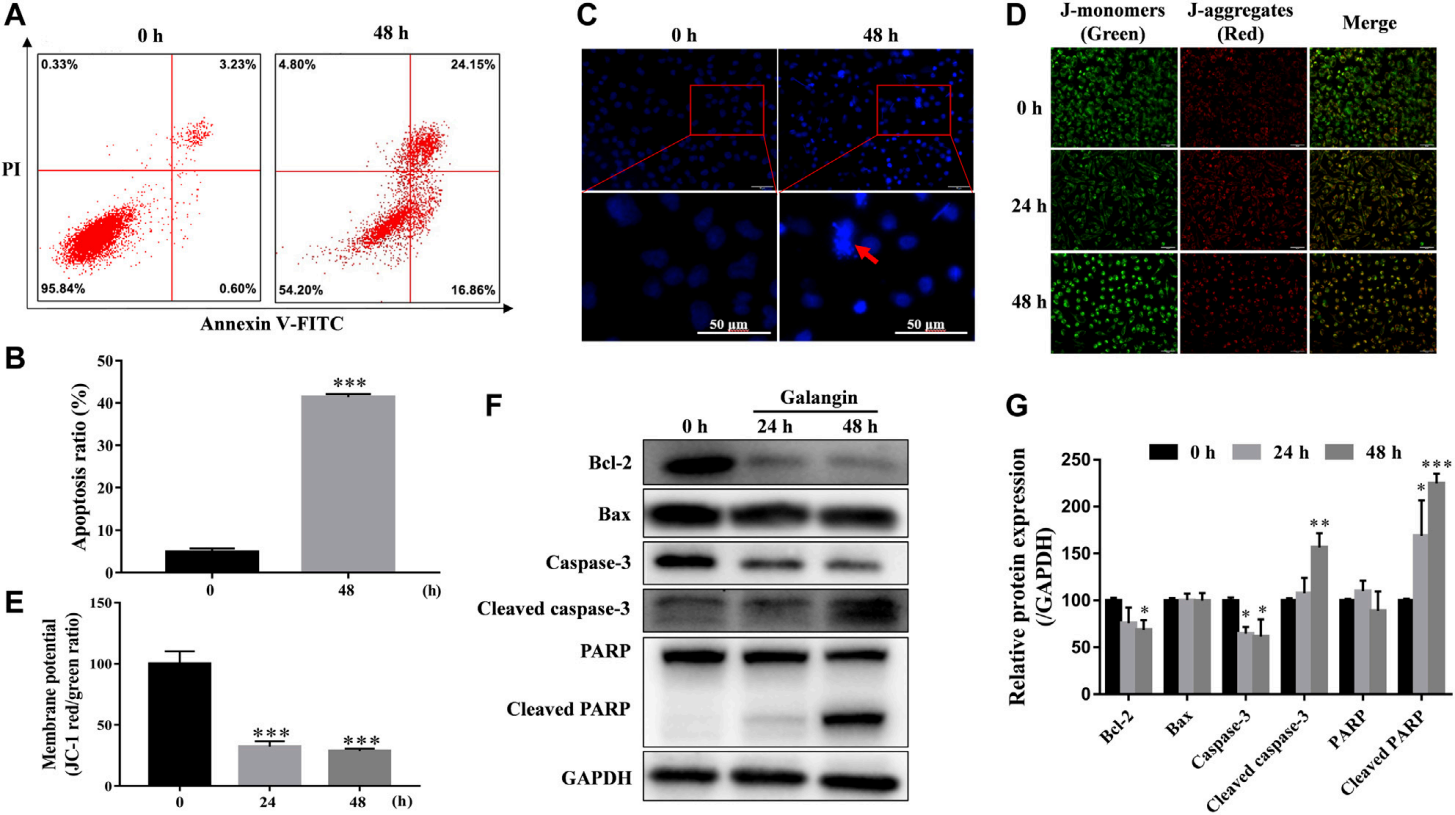
Galangin promotes cell apoptosis. **(A–B)** Glangin enhanced cell apoptosis after treatment for 24 h in MGC 803 cells detected by Annexin V-FITC/PI staining. **(C)** Galangin (20 μM) induced chromatin condensation and nuclear shrinkage or fragmentation in MGC 803 cells after 48 h treatment stained with Hoechst 33,258. **(D–E)** Galangin (20 μM) treatment decreased mitochondrial membrane potential (MMP) in MGC 803 cells after treatment for 24 and 48 h, detected by JC-1 (10 μM) staining. The staining of MGC 803 cells by JC-1 is visible as green for J-monomers (emission maximum of ∼529 nm) or red for J-aggregates (with a specific red fluorescence emission maximum at 590 nm). **(F–G)** Galangin (20 μM) modulated protein expression of apoptosis-related proteins in MGC 803 cells after treatment for 24 and 48 h analyzed by Western blotting analysis. All of the data were shown as mean ± SD, and differences among two groups (Figure 2B) were analyzed by the Student’s *t*-test, and differences among ≥3 groups (Fig.2E and G) were analyzed via one-way ANOVA with Dunnett test by using GraphPad 7.0 software; *p < 0.05; **p < 0.01; ***p < 0.001, compared with control group. n ≥ 3. Scale bar: 50 μm.

As shown in Figures 2A,B, compared with the untreated cells, galangin (20 μM) prominently increased the percentages of the early and late apoptotic cells by 41.41% after treatment for 48 h (*p* < 0.001). And it significantly induced chromatin condensation and nuclear shrinkage or fragmentation in MGC 803 cells (Figure 2C). MMP decrease is an early manifestation of cell apoptosis. Compared with the control, galangin significantly reduced the density ratio of JC-1 red/green fluorescence in MGC 803 cells, suggesting that galangin decreased MMP after treatment for 24 and 48 h (Figures 2D,E, *p* < 0.001). As shown in Figures 2F,G, after treatment for 48 h, compared with the control, galangin significantly decreased the protein expression of Bcl-2 and caspase-3, and up-regulated the protein expression of cleaved caspase 3 and cleaved poly (adenosine diphosphate-ribose) polymerase (PARP) (*p* < 0.05, *p* < 0.01 or *p* < 0.001). However, galangin did not change the expression of Bax in MGC 803 cells. These results indicated that galangin induced significant apoptosis of MGC 803 cells.

### Galangin Inhibited Proliferation of MGC 803 Cells

**FIGURE 3:**
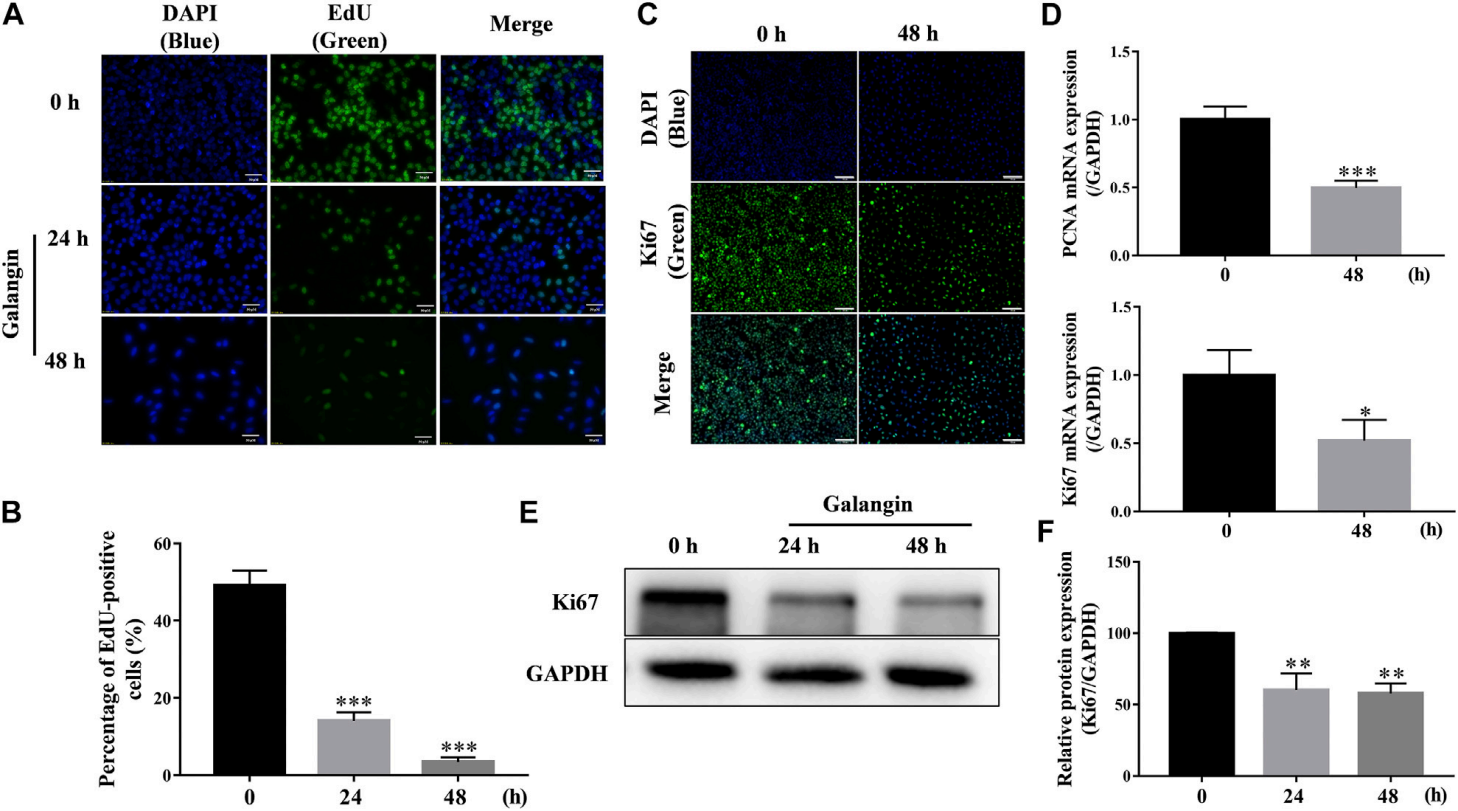
Galangin inhibits proliferation of MGC 803 cells. **(A–B)** Galangin (20 μM) decreased cell proliferation of MGC 803 cells after treatment for 24 and 48 h detected by EdU staining. Scale bar, 50 μm. **(C)** Galangin (20 μM) inhibited protein expression of Ki67 in MGC 803 cells detected by ICC staining. Scale bar, 100 μm. **(D)** Galangin (20 μM) downregulated mRNA expression of PCNA and Ki67 after treatment for 48 h. **(E–F)** Galangin (20 μM) decreased protein expression of Ki67 in MGC 803 cells after treatment for 24 and 48 h analyzed by Western blotting analysis. All of the data were shown as mean ± SD, and differences among two groups (Figure 3D) were analyzed by the Student’s *t*-test, and differences among ≥3 groups (Figures 3B,F) were analyzed via one-way ANOVA with Dunnett test by using GraphPad 7.0 software; *p < 0.05; **p < 0.01; ***p < 0.001, compared with control group. n ≥ 3.

As shown in Figures 3A,B, after treatment for 24 and 48 h, galangin significantly decreased the number of EdU-positive cells (*p* < 0.001), compared with the control. Ki67, a nuclear antigen, is a marker for cell proliferation. As shown in Figure 3C, compared with the control, galangin reduced the immuno-fluorescent intensity of Ki67 in MGC 803 cells. Meanwhile, galangin significantly inhibited the mRNA expression of PCNA, and suppressed the expression of Ki67 at both mRNA and protein levels (Figures 3D–F, *p* < 0.05, *p* < 0.01 or *p* < 0.001). These results implicated that galangin could inhibit the proliferaton of MGC 803 cells.

### Galangin Inhibited Cell Viability by Suppressing STAT3 Activation in MGC 803 Cells

**FIGURE 4:**
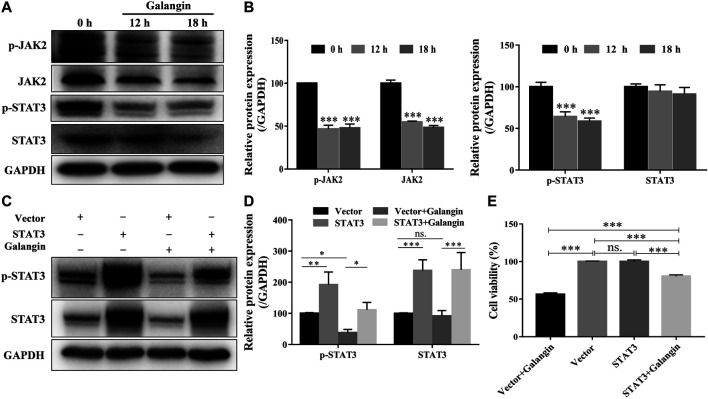
Galangin inhibits overactivation of JAK2/STAT3 signaling pathway. **(A–B)** Galangin (20 μM) decreased protein expression of JAK2, p-JAK2, STAT3, and p-STAT3 in MGC 803 cells after treatment for 12 and 18 h. **(C–D)** Overexpression of STAT3 reversed the inhibitory effect of galangin (20 μM) on the protein expression of p-STAT3 and STAT3 in MGC 803 cells after treatment for 48 h. **(E)** Overexpression of STAT3 reversed the inhibitory effect of galangin (20 μM) on the cell viability of MGC 803 cells after treatment for 48 h. All of the data were shown as mean ± SD, and differences among ≥3 groups were analyzed via one-way ANOVA with Dunnett test (Figure 4B, compared with control (0 h) group) or Tukey test (Figures 4D,E) by using GraphPad 7.0 software; *p < 0.05; **p < 0.01; ***p < 0.001. n ≥ 3.

As displayed in Figures 4A,B, galangin reduced the protein expression of *p*-JAK2, and p-STAT3 after treatment for 12 and 18 h in MGC 803 cells. When STAT3 was overexpressed, the inhibitory effect of galangin on the protein expressions of p-STAT3 and STAT3 was abolished (Figures 4C,D). Meanwhile, the cell viability inhibited by galangin on MGC 803 cells was counteracted by the overexpression of STAT3 (Figure 4E, *p* < 0.001). These results indicated that galangin inhibited the cell viability of MGC 803 cells through modulating the activation of STAT3.

### STAT3 Overexpression Counteracted Apoptosis Induced by Galangin in MGC 803 Cells

**FIGURE 5:**
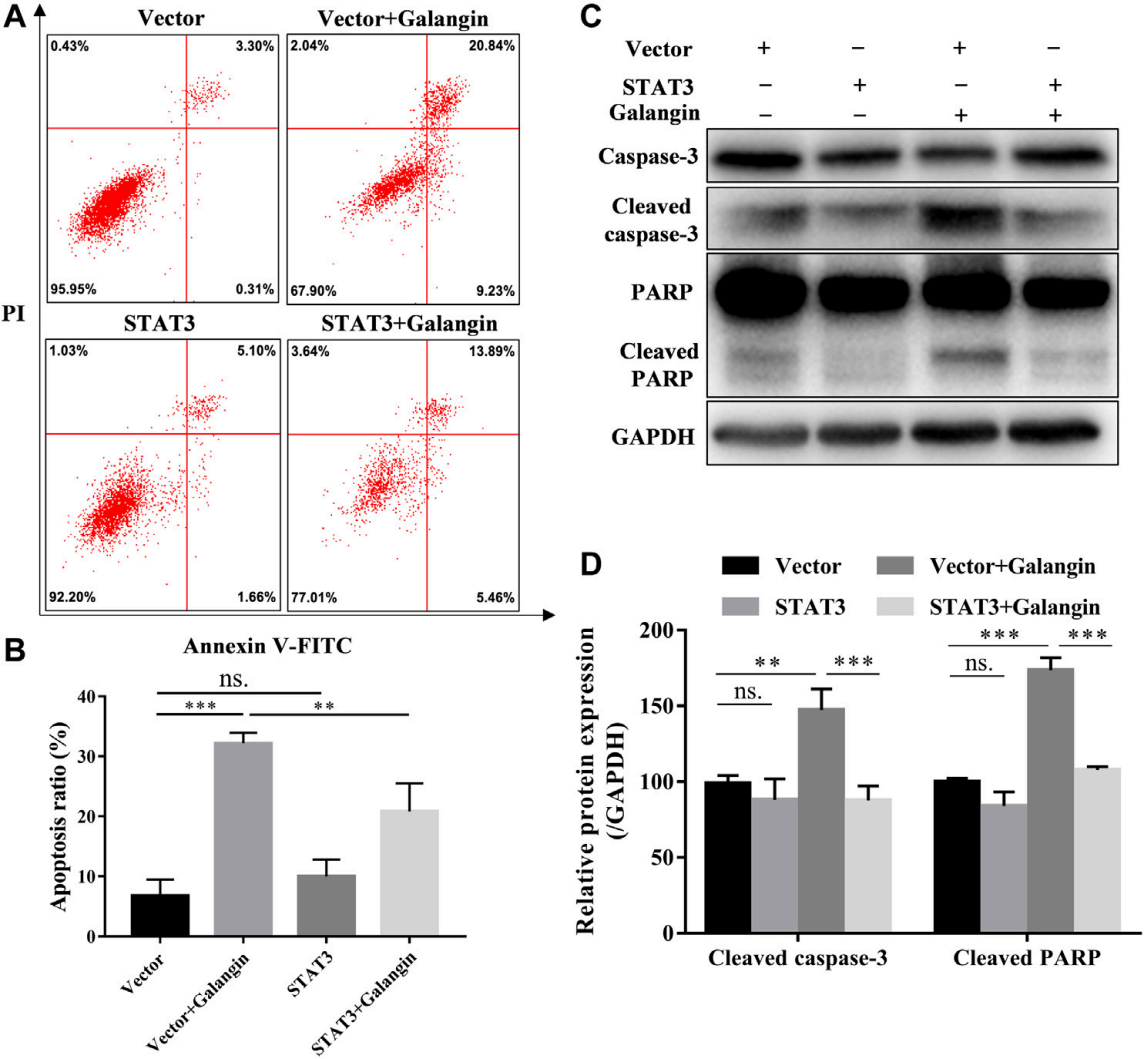
STAT3 overexpression counteractes the effect of galangin on cell apoptosis in MGC 803 cells. **(A–B)** STAT3 overexpression abolished the effect of galangin (20 μM) on cell apoptosis of MGC 803 cells after treatment for 48 h, detected by Annexin V/PI staining. **(C–D)** Overexpression of STAT3 reversed the effect of galangin (20 μM) on protein expression of cleaved caspase-3 and cleaved PARP in MGC 803 cells. All of the data were shown as mean ± SD, and differences among ≥3 groups (Figures 5B,D) were analyzed by one-way ANOVA with Tukey test by using GraphPad 7.0 software; **p < 0.01; ***p < 0.001. n ≥ 3.

To confirm the role of STAT3 in the effect of galangin on cell apoptosis, the STAT3 overexpressed cells treated with galangin were subjected to Annexin V/PI staining. As shown in Figures 5A,B, overexpression of STAT3 significantly mitigated the ratio of apoptotic cells induced by galangin in MGC 803 cells (*p* < 0.01). Furthermore, STAT3 overexpression counteracted the inductive effect of galangin on the protein expression of cleaved caspase-3 and cleaved PARP in MGC 803 cells after treatment for 48 h (Figures 5C,D). These results clearly clarified the essential role of STAT3 in galangin induced apoptosis of MGC 803 cells.

### Overexpression of STAT3 Reduced the Inhibitory Effect of Galangin on Cell Proliferation of MGC 803 Cells

**FIGURE 6:**
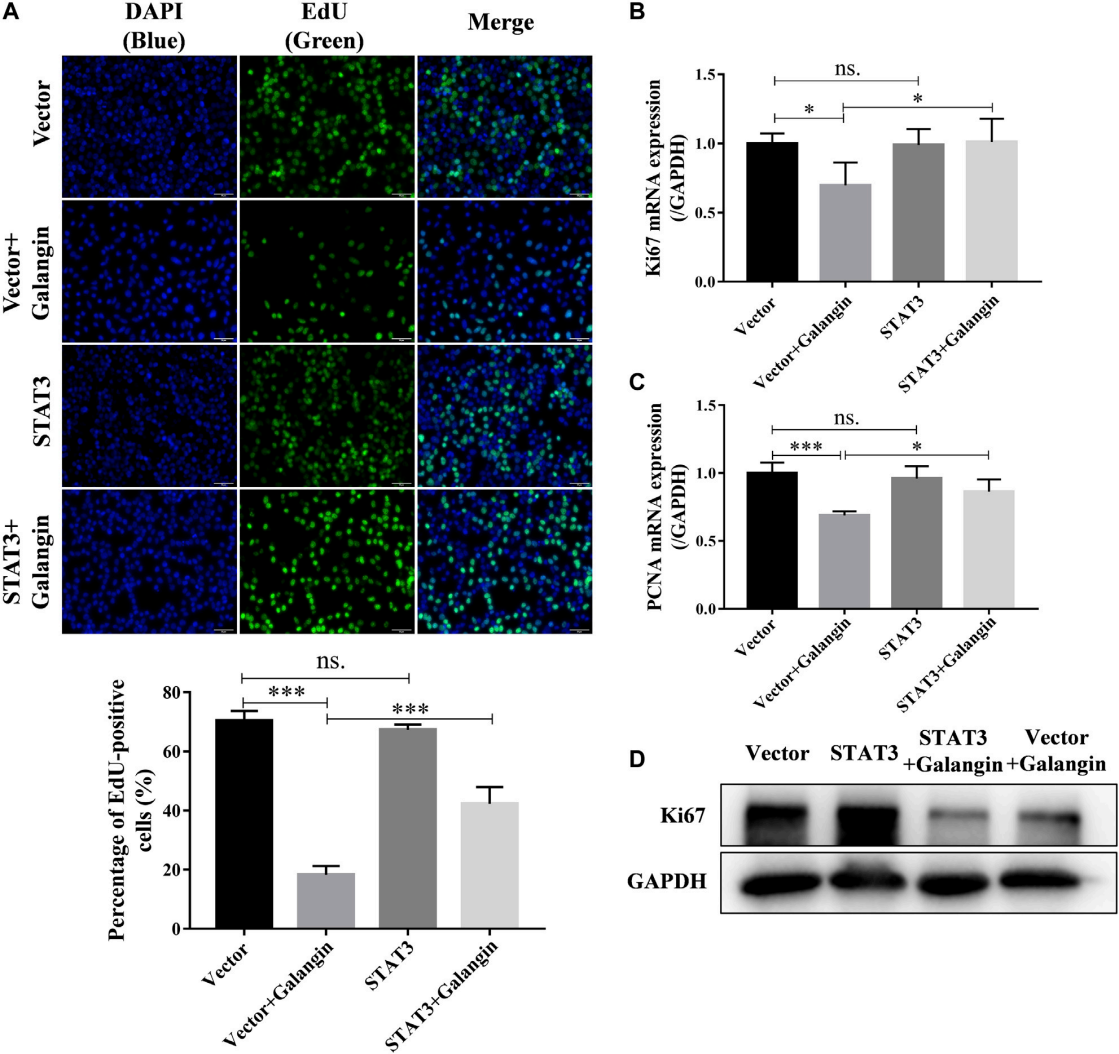
Overexpression of STAT3 counteractes the inhibition of galangin (20 μM) on cell proliferation of MGC 803 after treatment for 48 h. **(A)** STAT3 overexpression counteracted the inhibitory effect of galangin (20 μM) on cell proliferation of MGC 803 cells after treatment for 48 h, detected by EdU staining. **(B–C)** Overexpression of STAT3 reversed the effect of galangin on the mRNA expression of PCNA and Ki67 after treatment for 48 h in MGC 803 cells detected by qPCR assay. **(D)** Overexpression of STAT3 reversed the effect of galangin on protein expression of Ki67 in MGC 803 cells after treatment for 48 h. All of the data were shown as mean ± SD, and differences among ≥3 groups (Figures 6A–C) were analyzed by one-way ANOVA with Tukey test by using GraphPad 7.0 software; *p < 0.05; ***p < 0.001. n ≥ 3. Scale bar, 50 μm.

To confirm the role of STAT3 in the inhibition effect of galangin on cell proliferation, qPCR assay, Western blot method, and EdU staining were used to examine the effect of galangin on cell proliferation in STAT3 overexpressed MGC 803 cells. As shown in Figure 6A, overexpression of STAT3 markedly increased the pencentage of EdU positive cells treated by galangin (*p* < 0.001). Furthermore, STAT3 overexpression also reversed the inhibitory effect of galangin on the mRNA expression of PCNA and Ki67 as well as the protein expression of Ki67 (Figures 6B–D).

### Galangin Enhanced ROS Accumulation in MGC 803 Cells

**FIGURE 7:**
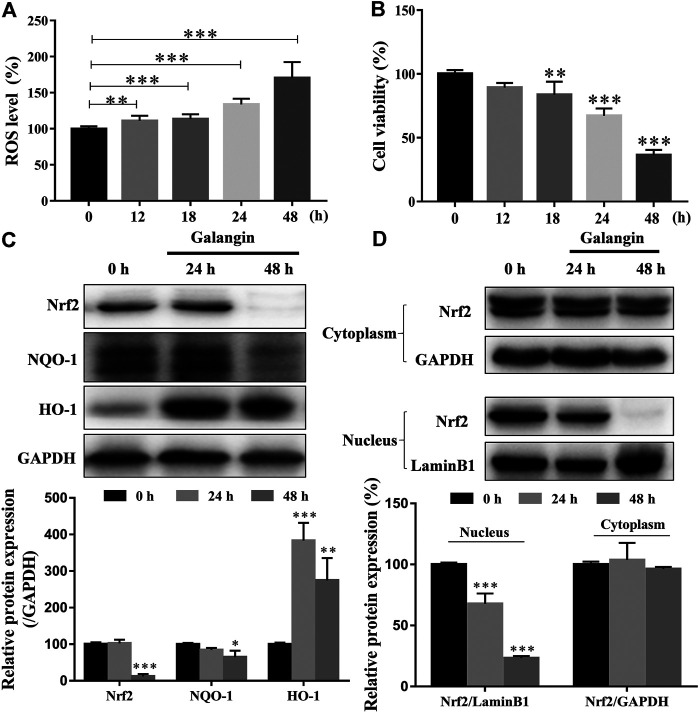
Galangin enhances ROS accumulation and reduces activation of Nrf2-mediated antioxidant system in MGC 803 cells. **(A–B)** Galangin (20 μM) promoted ROS levels in MGC 803 cells after treatment for 12, 18, 24, and 48 h. **(C)** Galangin (20 μM) reduced protein expression of Nrf2 and NQO-1, and enhanced protein expression of HO-1 after treatment for 48 h in MGC 803 cells. **(D)** Galangin (20 μM) inhibited Nrf2 translocation into nucleus of MGC 803 cells after treatment for 48 h. All of the data were shown as mean ± SD, and differences among ≥3 groups were analyzed by one-way ANOVA with Dunnett test by using GraphPad 7.0 software; **p < 0.01; ***p < 0.001, compared with control group. n ≥ 3.

As shown in Figure 7A, as early as 12 h after treatment, galangin (20 μM) significantly increased the ROS level in a time-dependent manner in MGC 803 cells. However, significantly decreased cell viability was observed after treated with galangin after 18 h (Figure 7B). In MGC 803 cells treated with galangin for 48 h, the protein expression of NF-E2-related factor 2 (Nrf2) and NAD(*p*)H quinone oxidoreductase 1 (NQO-1) were reduced, while the protein expression of heme oxygenase-1 (HO-1) was elevated (Figure 7C). Furthermore, the translocation of Nrf2 into nucleus was reduced significantly after treated by galangin for 48 h (Figure 7D). These results indicated that over-produced ROS and dysfunctioned antioxidant system might finally account for the inhibitory effect of galangin on the growth of MGC 803 cells.

### STAT3/ROS *Axis* Mediated the Inhibition of Galangin on Cell Proliferation of MGC 803 Cells

**FIGURE 8:**
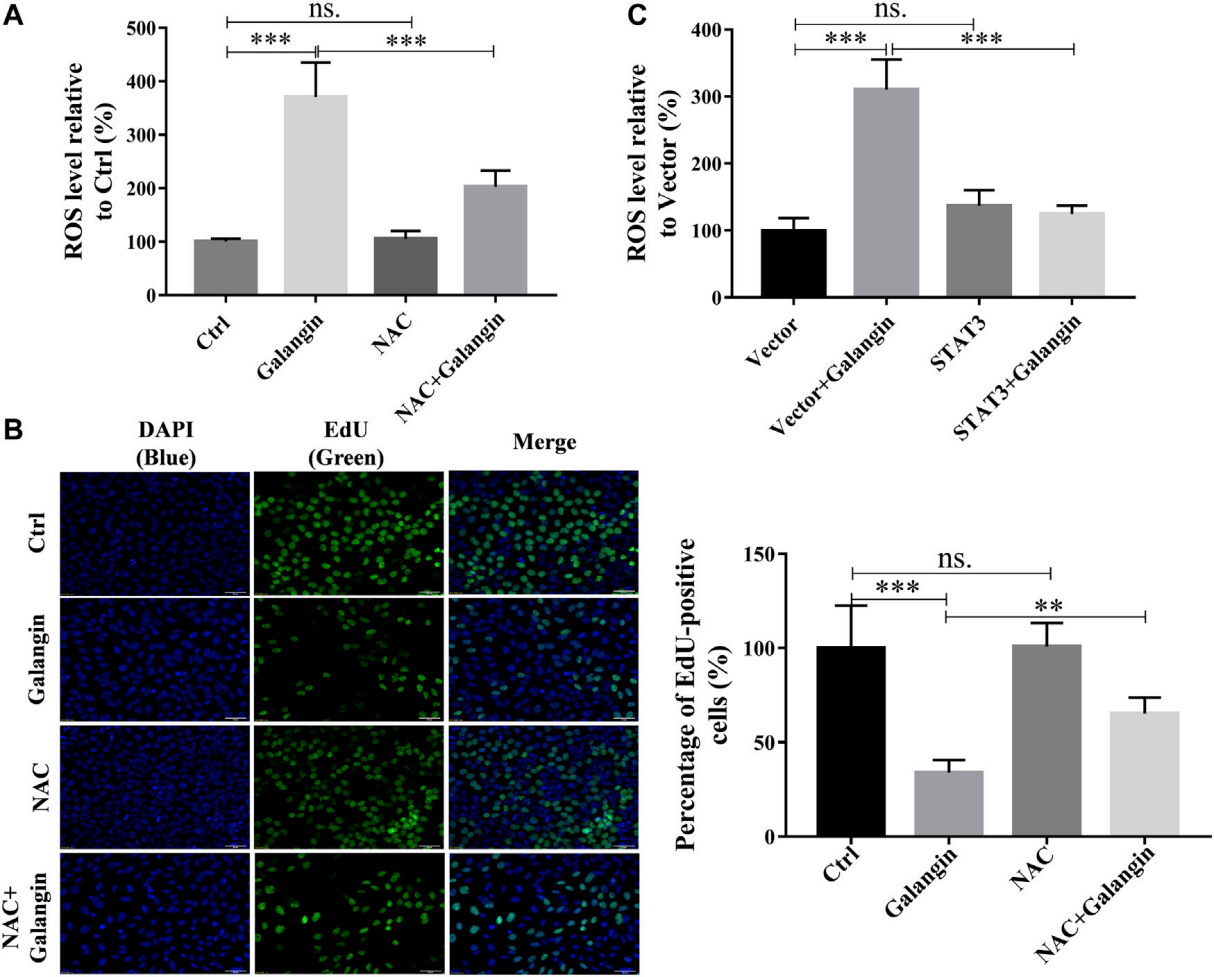
The inhibition of galangin on cell proliferation is associated with ROS generation. **(A)** NAC (ROS scavenger) abolished the enhanced ROS accumulation in MGC 803 cells induced by galangin (20 μM) after treatment for 48 h. **(B)** NAC counteracted the inhibited cell proliferation induced by galangin (20 μM) after treatment for 48 h detected by EdU staining in MGC 803 cells. **(C)** STAT3 overexpression reversed the enhanced ROS accumulation in MGC 803 cells induced by galangin (20 μM) after treatment for 48 h. All of the data were shown as mean ± SD, and differences among ≥3 groups were analyzed by one-way ANOVA with Tukey test by using GraphPad 7.0 software; **p < 0.01; ***p < 0.001. n ≥ 3. Scale bar: 50 μm.

To explore the possible role of the production of ROS in galangin-treated MGC 803 cells, NAC, a ROS scavenger, was used. As displayed in Figure 8A, addition of NAC markedly reduced ROS level in galangin treated MGC 803 cells (*p* < 0.001). Correspondingly, compared with cells treated with only galangin, the percentage of EdU positive cells were increased significantly in NAC and galangin co-treated cells (Figure 8B, *p* < 0.01 or *p* < 0.001). To further investigate the relationship between STAT3 activation and ROS production, the ROS level in STAT3 overexpressed cells was examined. As shown in Figure 8C, overexpression of STAT3 did not change the ROS production in MGC 803 cells. However, STAT3 overexpression reduced the ROS accumulation induced by galangin treatment in cells (Figure 8C, *p* < 0.001). These results suggested that galangin suppressed the activation of STAT3, thereby increased the generation of ROS, finally leading to the decrease of cell proliferation of gastric cancer cells.

### Galangin Inhibited Tumor Growth *In Vivo*

**FIGURE 9:**
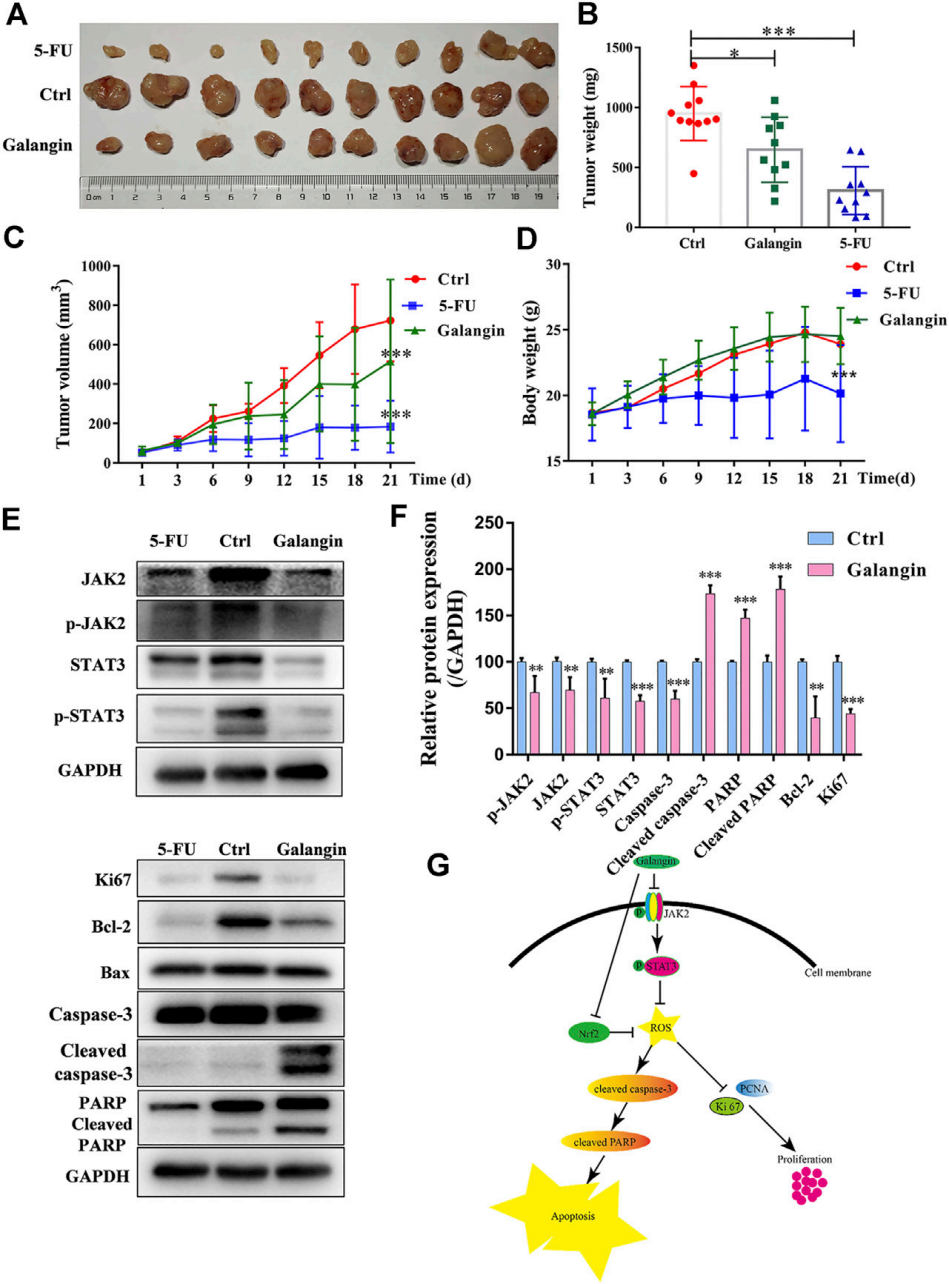
Galangin inhibits tumor growth in the MGC 803 cell xenograft model. **(A–B)** Galangin (120 mg/kg) significantly inhibited tumor growth (*n* = 10). **(C)** Galangin (120 mg/kg) significantly inhibited tumor volume (*n* = 10). **(D)** Body weight changes (n = 10). **(E-F)** Galangin modulated protein expression of JAK2, p-JAK2, STAT3, p-STAT3, Bcl-2, Caspase-3, Cleaved caspase-3, PARP, Cleaved PARP, and Ki67 in tumor tissues (*n* = 6). **(G)** Schematic illustration of possible underlying molecular mechanism of the inhibitory effect of galangin on MGC 803 cells. All of the data were shown as mean ± SD, and differences among ≥3 groups (Figures 9B–F) were analyzed by one-way ANOVA with Dunnett test by using GraphPad 7.0 software; *p < 0.05, **p < 0.01; ***p < 0.001, compared with control group.

To verify the inhibitory effect of galangin on gastric cancer growth *in vivo*, the MGC 803 cell xenograft mouse model was established. Compared with the control, galangin and 5‐FU treatment significantly inhibited the increase of tumor weight and volume *in vivo* (Figures 9A–C, *p* < 0.05 or *p* < 0.001). Nevertheless, as shown in Figure 9D, galangin showed no significant effect on body weight of nude mice. In contrast, 5-FU significantly decreased the body weight of nude mice (*p* < 0.001). In tumor tissues from galangin treated mice, the ratios of *p*-JAK2/JAK2 and p-STAT3/STAT3, as well as the protein expressions of Bcl-2, caspase-3 and Ki67 were all reduced remarkably (Figures 9E,F, *p* < 0.01 or *p* < 0.001). Conversely, the protein expressions of cleaved caspase-3 and cleaved PARP were increased markedly by galangin treatment (*p* < 0.001). These results suggested that galangin could inhibit gastric cancer growth *in vivo*.

## Discussion

Flavonoids are widely distributed in nature, and often can be found in food. Galangin, a natural flavonoid compound mainly present in the rhizome of *Alpinia officinarum* Hance (Zingiberaceae) (Liu et al., 2018), shows extensive anti-tumor activities except in gastric cancer. Impaired proliferation and apoptosis commonly account for the cancer progression (Goldar et al., 2015; White, 2015). In the present study, galangin was found to significantly suppress the proliferation, while induced the apoptosis of MGC 803 cells both *in vitro* and *in vivo.* Moreover, it showed no toxicity on normal gastric mucosal epithelial cell line (GES-1 cells) and induced no body weight loss in nude mice, suggesting its potential clinical application for the treatment of gastric cancer possibly with low toxicity.

STAT3 dysfunction accounts for the impaired cell proliferation and apoptosis in cancer cells (Fathi et al., 2018; Liang and Yang, 2020). JAK-medicated tyrosine phosphorylation enhances the dimerization of STATs. In the present study, galangin reduced the phosphorylated JAK2 and STAT3 in MGC 803 cells at 12 h, and the inhibitory effect of galangin on cell viability of MGC 803 cells was counteracted by STAT3 overexpression, indicating galangin inhibited gastric cancer in a STAT3-dependent manner.

Mitochondrial-dependent apoptosis plays an important role in cell death. Bcl-2 inhibits cell apoptosis, however, Bax promotes cell apoptosis (Mao et al., 2007). Both activated caspase-8 and caspase-9 can activate caspase-3 which is the central link of apoptosis (Zheng et al., 2006). Then caspase-3 cleaves several cellular proteins, including PARP, causing morphological changes and DNA breaks, and ultimately leading to apoptosis (Zheng et al., 2006; Yang et al., 2017). In the present study, galangin significantly decreased Bcl-2 and increased cleaved caspase-3 and cleaved PARP. STAT3 overexpression counteracted the enhanced apoptosis induced by galangin, and reversed the inductive effect of glangin on cleaved caspase-3 and cleaved PARP, indicating that galangin induced apoptosis in a STAT3-dependent manner through enhancing cleavage of caspase-3 and its downstream PARP in MGC 803 cells. Meanwhile, STAT3 overexpression abolished the inhibitory effect of galangin on cell proliferation as well as Ki67 and PCNA expression, indicating galangin inhibited cell proliferation also in a STAT3-dependent manner.

Low concentration of ROS can activate transcription factors to promote cell proliferation and differentiation, but excessive ROS can induce depolarization of the mitochondrial membrane, thereby promote the increase of other pro-apoptotic molecules in the cells, reduce the proliferation and survival of tumor cells, and promote cell apoptosis (Leanza et al., 2017; Prasad et al., 2017). In the present study, galangin increased intracellular ROS accumulation of MGC 803 cells in a time-dependent manner. Meanwhile, Nrf2-mediated antioxidant system was significantly decreased, as evidenced by the decreased Nrf2 and NQO-1 in MGC 803 cells. Furthermore, galangin also suppressed Nrf2 translocation into nucleus, indicating galangin increased ROS level by suppressing Nrf2 mediated antioxidant system. NAC could abolish galangin-induced ROS accumulation and block the inhibitory effect of galangin on cell proliferation, indicating galangin suppressed cell proliferation in a ROS-dependent manner. Furthermore, STAT3 overexpression almost completely abolished the ROS accumulation induced by galangin treatment, indicating galangin induced ROS generation through STAT3 suppression. Many researches have proved that ROS enhanced cell apoptosis (Cui et al., 2018; Dong et al., 2019), however, it has not been found that STAT3 also modulates ROS levels. Our results demonstrated that STAT3 activation inhibited ROS overload. Therefore, galangin might inhibit cell proliferation and enhance apoptosis by modulating STAT3/ROS axis in MGC 803 cells. 5-FU is a first-line chemotherapeutic drug for gastric cancer in clinic, that’s why we choose 5-FU as the positive drug in our study. However, it has serious adverse effects including bone marrow suppression, digestive tract toxicity and drug resistance (Wang et al., 2020). Galangin showed no obvious cytotoxicity on normal gastric mucosal epithelial cell line, GES-1 cells. In contrast, 5-FU (≥10 μM) significantly inhibited cell viability of GES-1 cells. Body weight loss is an important indicator of *in vivo* toxicity (Cai et al., 2020). Galangin did not reduce the body weight of nude mice. However, 5-FU showed the negative effect. Thus, galangin inhibited gastric tumor growth possibly with low toxicity. However, the low toxicity and its underlying molecular mechanism of galangin on normal cells still need further study.

In summary, galangin inhibited MGC 803 cells growth through enhancing apoptosis and decreasing cell proliferation, which was mediated by modulating STAT3/ROS axis. Our findings suggest that galangin is a potential drug for gastric cancer treatment with possibly low toxicity.

## Data Availability

The original contributions presented in the study are included in the article/Supplementary Material, further inquiries can be directed to the corresponding authors.

## Abbreviations

DCFH-DA, 2′,7′-dichlorofluorescin diacetate; JAK2, Janus associated kinase; HIF1α, hypoxia-inducible factor 1α; HO-1, heme oxygenase-1; MMP, Mitochondrial membrane potential; NF-κB, nuclear factor-kappa B; NQO1, NAD(P)H quinone oxidoreductase 1; Nrf2, NF-E2-related factor 2; PARP, poly (adenosine diphosphate-ribose) polymerase; ROS, reactive oxygen species; STAT3, signal transducer and activator of transcription 3; 5-FU, 5-Fluorouracil.
